# Obituary

**Published:** 1911-10-14

**Authors:** 


### Obituary.

The death of Surgeon-General C. K. Colston, I.M.S. (retired), took place recently at Teignmouth. General Colston since his retirement had taken an interest in local education affairs, and was for many years a member of the old School Board for the town of Teignmouth.

The death is announced of Surgeon-Major John Raby, I.M.S., who died at Paignton from an overdose of an hypnotic. In the hunting-field he was well known, especially in the neighbourhood of Taunton, where he formerly lived, and he frequently figured as a successful competitor at the steeplechase meetings in the West of England. Major Raby graduated at Edinburgh and London in 1865. He received official recognition from the Indian Government for his active services in the Abyssinian expedition.

The death is announced of Dr. Samuel Mills, of Portland Place, W., in his seventy-first year. Dr. Mills was honorary surgeon to the Corps of Commissionaires, to whom he was for a long time surgeon. He studied at University College, and qualified in 1863. His former appointments include those of resident physician's assistant at Brompton Hospital for Consumption, and surgeon to the Bow Street Division of the Metropolitan Police.

The death is announced of Dr. Albert Wheeler, formerly of East Dulwich, in his sixty-fifth year. Dr. Wheeler was a former student of the Middlesex Hospital, and qualified in 1879. He held the appointment of house physician at his own hospital, and later took the M.D. degree of the University of St. Andrews. For very many years he was one of the best-known practitioners in the Honor Oak Park district.

The death has occurred at his residence in Bransgore, Hants, of Dr. Leonard Keatley Yelf, in his eighty-fourth year. Dr. Yelf studied at King's College, London, and qualified as far back as 1852. He afterwards took the M.D. of St. Andrews University. Dr. Yelf was for a long time surgeon to the Moreton Cottage Hospital.

